# Developmental endothelial locus 1: the present and future of an endogenous factor in vessels

**DOI:** 10.3389/fphys.2024.1347888

**Published:** 2024-08-09

**Authors:** Daisong Jiang, Honghua Yue, Wei-Tao Liang, Zhong Wu

**Affiliations:** Department of Cardiovascular Surgery, West China Hospital, Sichuan University, Chengdu, Sichuan, China

**Keywords:** developmental endothelial locus-1, integrin, inflammation, efferocytosis, vascular smooth muscle cell

## Abstract

Developmental Endothelial Locus-1 (DEL-1), also known as EGF-like repeat and discoidin I-like domain-3 (EDIL3), is increasingly recognized for its multifaceted roles in immunoregulation and vascular biology. DEL-1 is a protein that is mainly produced by endothelial cells. It interacts with various integrins to regulate the behavior of immune cells, such as preventing unnecessary recruitment and inflammation. DEL-1 also helps in resolving inflammation by promoting efferocytosis, which is the process of clearing apoptotic cells. Its potential as a therapeutic target in immune-mediated blood disorders, cardiovascular diseases, and cancer metastasis has been spotlighted due to its wide-ranging implications in vascular integrity and pathology. However, there are still unanswered questions about DEL-1’s precise functions and mechanisms. This review provides a comprehensive examination of DEL-1’s activity across different vascular contexts and explores its potential clinical applications. It underscores the need for further research to resolve existing controversies and establish the therapeutic viability of DEL-1 modulation.

## 1 Introduction

Increasing evidence suggests that local tissues, which were previously thought to be passive recipients of immunity, have equally active regulatory functions (Galli et al., 2011). The requirements of the tissues to which immune cells are recruited or reside are constantly changing, and the function of these cells is constantly being adapted (Galli et al., 2011; Matzinger and Kamala, 2011; Oyler-Yaniv et al., 2017). Immune plasticity can manifest at different levels, from the cellular to the molecular level (Galli et al., 2011). Cellular plasticity refers to the ability of cells to adapt and change their characteristics or functions in response to various stimuli or environmental cues (Oyler-Yaniv et al., 2017; Hajishengallis and Chavakis, 2019).

**FIGURE 1:**
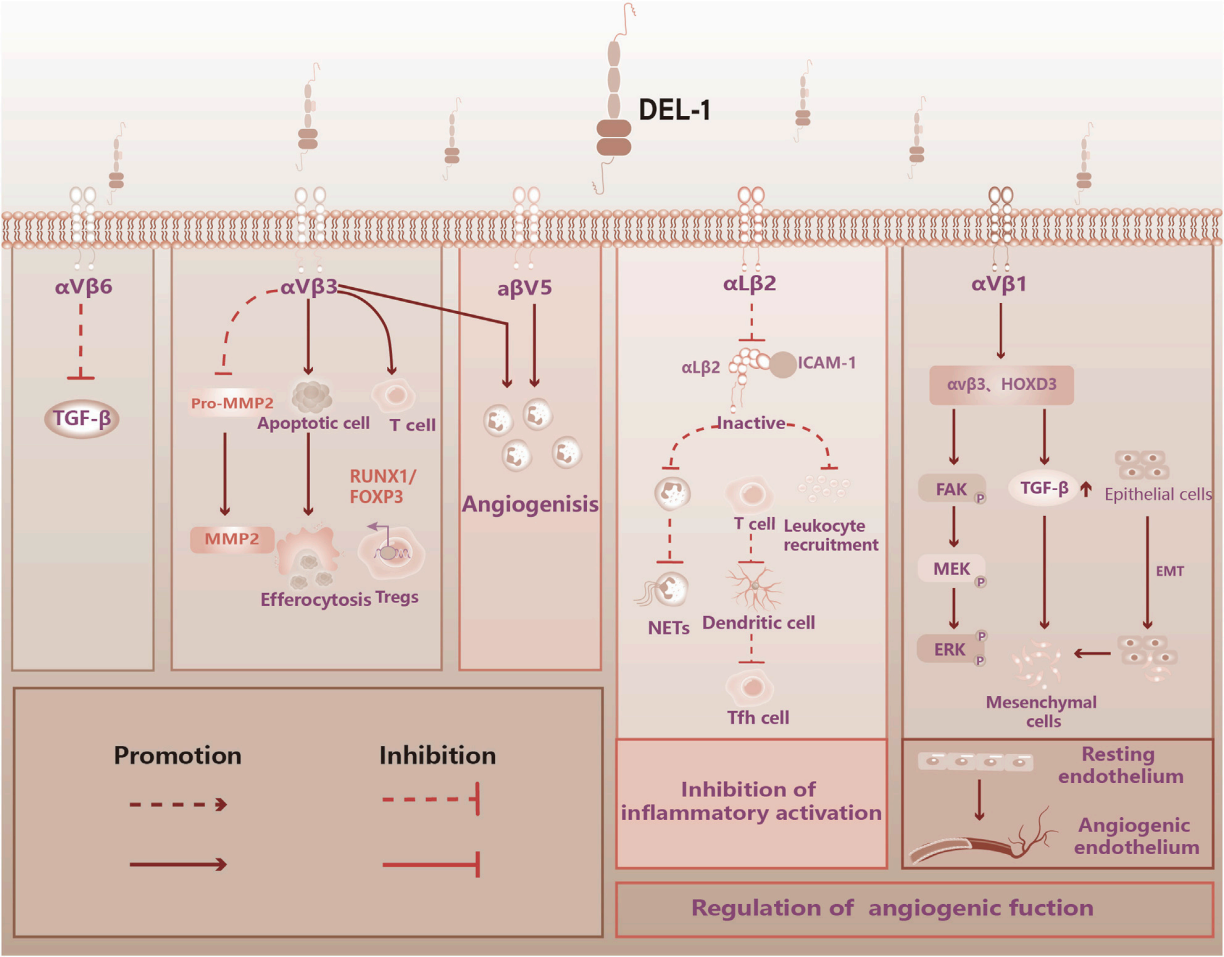
DEL-1 binds to integrin to perform immune functions in the vasculature. Figure 1 illustrates the physiological functions of DEL-1, which are known to bind to some of the integrins. DEL-1 binds to αvβ3 integrin to inhibit TGF-β activation. DEL-1 binds to αvβ3 integri: Inhibits the production of MMP2 by MMP2 precursors; Promotes phagocytosis of apoptotic cells by phagocytosis; Stabilized αvβ3 integrin-dependent Treg number; Promoting angiogenic effects. DEL-1 binding to αvβ5 integrin: Promoting angiogenic effects. DEL-1 binds to αLβ2 to inhibit inflammatory effects: Inhibition of Leukocyte aggregation; Inhibition of neutrophil release of neutrophil extracellular traps (NETs); Inhibition of T cell conversion to Tfh cell. DEL-1 binds to αvβ1: regulation of the corresponding signaling pathway promotes the transformation of resting endothelium into angiogenic endothelium. TGF-β: Transforming Growth Factor-beta, FOXP3: Forkhead Box Protein P3, NETs: Neutrophil Extracellular Traps, HOXD3: Homeobox D3, FAK: Focal Adhesion Kinase, MEK: Mitogen-Activated Protein Kinase Kinase, ERK: Extracellular Signal-Regulated Kinase, EMT: Epithelial-Mesenchymal Transition.

DEL-1 is a 52 kDa protein encoded by the EDIL3 gene (C Hidai 1, 1998). DEL-1 contains three epidermal growth factor (EGF)-like repeats at the N-terminus and the Arg-Gly-Asp (arginine-glycine-aspartate, RGD) module in the second EGF domain (EGF2) which combines with integrin to form a recognition system for cell adhesion (Ruoslahti, 1996; Schurpf et al., 2012). At the C-terminus, DEL-1 has two discoidal I-like domains capable of glycosaminoglycan and phosphatidylserine interactions (Hanayama et al., 2004; Hidai et al., 2007). DEL-1 is released from endothelial cells, mesenchymal stromal cells, and subsets of macrophages (Hajishengallis and Chavakis, 2019). DEL-1, due to its unique structure, can interact with a variety of integrin types, including Macrophage-1 antigen (Mac-1, αMβ2 integrin), lymphocyte function-associated antigen 1 (LFA-1, αLβ2 integrin), αvβ3 integrin, αvβ6 integrin (Choi, 2009; Mitroulis et al., 2014; Kim et al., 2020; Li et al., 2021a). Its presence suggests that local factors regulate immune plasticity within tissues to maintain or reinstate tissue homeostasis (see Figure 1).

## 2 DEL-1 in immune modulation and inflammation

### 2.1 Structural and functional analysis of DEL-1

Integrins are versatile transmembrane receptors that function primarily as cell adhesion molecules, playing crucial roles in cellular processes including signaling, migration, and tissue integrity (Rose et al., 2007). Integrins modulate a range of physiological and pathological processes, including by promoting or inhibiting the inflammatory response, contributing to thrombosis through platelet aggregation and endothelial interactions, and affecting tissue remodeling during fibrosis (Mezu-Ndubuisi and Maheshwari, 2021; Mrugacz et al., 2021; Van Hove et al., 2021). DEL-1 serves as a ligand for integrins and also interacts with phospholipids, playing a role in various integrin-mediated processes, modulating different stages of the host inflammatory response (Hajishengallis and Chavakis, 2019; Hajishengallis and Chavakis, 2021). DEL-1 regulates immune cell functions, including leukocytes and effector cells (such as T cells), through integrins (Hajishengallis et al., 2015; Wang et al., 2021) (see Table 1).

**TABLE 1:** Important studies on integrin-mediated downstream immune function by DEL-1.

Type of Integrin	First author	Year	Subject (cell, participants, animals)	Main effect
α_L_β2 (LFA-1)	Wang H et al. (Wang et al., 2021)	2021	C57BL/6J mice	Inhibiting the induction of Tfh cells
	Yang N et al. (Yang et al., 2015)	2015	BMSCs / C57BL/6J mice	Transformation of Th17 cells to Treg
	Eskan MA et al. (Eskan et al., 2012)	2012	C57BL/6J mice	Inhibition of IL-17 mediated inflammation
	Yan S et al. (Yan et al., 2018)	2018	asthmatic patients,controls	
αMβ2(Mac-1)	Shin J et al. (Shin et al., 2015)	2015	C57BL/6J mice, cynomolgus monkeys	Inhibition the osteoclastogenesis
αvβ3	Kourtzelis I et al. (Kourtzelis et al., 2019)	2019	HMDMs, BMDMs, peritoneal macrophages, neutrophils, C57BL/6 mice	Enhances efferocytic activity
	Li X et al. (Li et al., 2020a)	2020	Human peripheral Treg, C57BL/6 mice	Reducing monocyte/macrophage and T cell infiltration
αvβ3/αvβ5	Zhong J et al. (Zhong et al., 2003)	2003	HUVEC,C57BL/6 mice, New Zealand white rabbits	Promotes angiogenesis
αvβ6	Kim DY et al. (Kim et al., 2020b)	2020	Primary lung fibroblasts, C57BL/6 mice	Hinders integrin-mediated TGF-β activation

MDMs: Human monocyte-derived macrophages, BMDMs; Mouse bone marrow-derived macrophages, HUVEC: Human umbilical vein endothelial cells.

### 2.2 DEL-1 combining integrins to regulate immune response and suppress inflammation

Endothelial cell-derived DEL-1 acts on integrins such as LFA-1, and Mac-1, interrupting the binding, to their respective ligands, thereby acting as an intrinsic regulator of leukocytes recruitment and a suppressor of early-stage inflammation (Mitroulis et al., 2014; Jia et al., 2023).

Previous studies have confirmed the anti-inflammatory properties of DEL-1 (Maekawa et al., 2020). Erythromycin-induced DEL-1 transcription attenuates LFA-1-mediated neutrophilic and mucosal inflammation (Maekawa et al., 2020). Additionally, DEL-1 transcription was mediated by Janus kinase 2 (JAK2) and the Phosphoinositide 3-kinase (PI3K)/Protein Kinase B (AKT) signaling pathway. The activation of these signaling pathways upregulates DEL-1 expression in lung microvascular endothelial cells Targeting these signaling pathways to enhance DEL-1 expression could offer a novel approach to treating inflammatory and age-related diseases (Maekawa et al., 2020). In a mouse model of colon cancer, DEL-1 was shown to reduce neutrophil recruitment and infiltration while increasing the number of M1 macrophages as an efficient anticancer immunotherapy (Tian et al., 2022).

Modulating EDIL3 gene expression has been shown to influence immune cells and cytokines and may contribute to various pathologies, including acute lung injury and fibrosis (Choi et al., 2008; Kim et al., 2020; Li et al., 2022b). In several mouse models of hypertension, Filer et al. discovered that while DEL-1 upregulation did not lead to differential expression of pro-metalloproteinase-2 (Pro-MMP-2) in the aorta and heart, it did inhibit αvβ3 integrin-dependent activation of Pro-MMP-2 in isolated mouse and human aortas. This inhibition prevented the transformation of Pro-MMP-2 to active MMP-2, significantly reducing MMP-2 expression (Failer et al., 2022).

Injection of recombinant DEL-1 protein has been observed to attenuate the increase in systolic blood pressure, likely through the prevention of adverse aortic remodeling, potentially by improving endothelial function and reducing endothelial permeability (Failer et al., 2022). Whereas DEL-1 deficiency has been correlated with heightened neutrophil infiltration and enhanced formation of neutrophil extracellular traps (NETs), thus exacerbating pressure-overload-induced heart failure. Conversely, *in vitro* experiments have revealed that DEL-1 can inhibit the formation of NETs in a Mac-1-dependent mechanism (Zhao et al., 2023).

### 2.3 DEL-1 regulates T cells’ functions

#### 2.3.1 DEL-1 interplay with Th17

IL-17, primarily released by Th17 cells, plays a crucial role in neutrophil recruitment, the production of the pro-inflammatory cytokines IL-1α, IL-1β, IL-6, and TNF, and host antimicrobial peptides, such as β-defensin 2 (Liang et al., 2006; Amatya et al., 2017; Altieri et al., 2018). IL-17 is involved in multiple autoimmune and cardiovascular diseases (Mills, 2023).

DEL-1 inhibits IL-17-induced immune cell recruitment and migration, effectively inhibiting inflammation associated with various diseases such as multiple sclerosis and bone loss (Eskan et al., 2012; Khader, 2012; Choi et al., 2015). Local administration of DEL-1 in the airways effectively curtails IL-17 production and diminishes neutrophil accumulation, reinforcing its role as an anti-inflammatory agent (Yan et al., 2018). DEL-1 exerts its inhibitory effects by targeting intercellular adhesion molecule 1 (ICAM-1)-dependent neutrophil recruitment, thereby mitigating airway inflammation (Choi et al., 2008). When the homeostatic regulation of DEL-1 and IL-17 is disrupted, it can drive associated inflammatory diseases when IL-17 is dominant (Hajishengallis and Chavakis, 2019). Previous studies have reported that IL-17 can downregulate DEL-1 in human endothelial cells by targeting the key transcription factor CCAAT/enhancer-binding protein (C/EBPβ) via glycogen synthase kinase-3 (GSK-3β), and this effect can be reversed by Resolvin-D1 (RvD1) (Maekawa et al., 2015). Recent research indicates that DEL-1 and RvD1 collaboratively impede inflammation. The RvD1-DEL-1 axis inhibits IL-17-induced pathological inflammation in a DEL-1-dependent manner. Upon interaction with G-protein-coupled receptor 32 (GPR32) and lipoxin A4 receptor/formylpeptide receptor 2 (ALX/FPR2) on endothelial cells, RvD1 facilitates anti-inflammatory actions (Maekawa et al., 2015).

DEL-1 deficiency leads to the overproduction of IL-17 and neutrophil migration, resulting in increased endothelial permeability and damage to the blood-brain barrier (Choi et al., 2015). In vascular diseases, various stimuli upregulate IL-17 expression (Nishida et al., 2020). In Kawasaki disease, high DEL-1 antibody levels are associated with excessive IL-17-mediated inflammation, and DEL-1 deficiency can exacerbate the pro-inflammatory response and increase the likelihood of coronary atherogenesis (Consiglio et al., 2020). IL-17 can eenhance WNT/β-catenin signaling, which exerts proliferative and anti-apoptotic effects on vascular smooth muscle cells (VSMCs) via this pathway (Zhang et al., 2018; Wang et al., 2019b). Th17/IL-17 can activate the downstream NF-κB pathway to induce endothelial cell senescence (Zhang et al., 2021). Given DEL-1’s role in the d downregulating IL-17 activity (Yan et al., 2018) its modulation presents a promising therapeutic avenue for the management of immune and inflammatory diseases characterized by aberrant IL-17 signaling.

#### 2.3.2 DEL-1 promotes T cell phenotype maintenance

Regulatory T cells, also known as Treg cells, are a crucial type of immune cell that helps in preventing autoimmune diseases and maintaining immune tolerance (Kim, 2009). These cells suppress the function of effector T cells which can cause harm to the body. Although it is not yet clear how Treg cells protect the cardiovascular system, their positive effects suggest that they could be a promising treatment option (Meng et al., 2016). The bone marrow serves as a reservoir for Treg cells (Zou et al., 2004). Notably, glucocorticoid (GC)-induced leucine zipper (GILZ) expression in bone marrow mesenchymal stem cells (MSCs) increases the expression and secretion of DEL-1 (Yang et al., 2015b). Additionally, DEL-1 can facilitate the transformation of Th17 cells to Treg cells via GILZ, thus stimulating the generation of Treg cells (Yang et al., 2015a). T cells are a diverse group of immune cells found in atherosclerotic plaques, which are the buildup of fat and other substances in artery walls. T cells are distributed differently in the intimal layer of atherosclerotic plaques (Libby, 2012; Marchini et al., 2021). The forkhead box transcription factor (FOXP3) is a transcription factor that plays a key role in the development and function of Treg cells (Hori et al., 2003).

DEL-1 stabilized αvβ3 integrin-dependent Treg number, which reduced inflammatory cell recruitment and cytokine production in cardiovascular organs (Failer et al., 2022). *In vivo*, FoxP3^+^ Treg cells can inhibit the development of atherosclerosis by exerting regulatory control over lipoprotein metabolism. Treg cell lineages demonstrate plasticity, and the downregulation or loss of FoxP3 expression in ApoB-reactive (ApoB+) Treg cells was observed during lineage tracing in ApoE-/- mice (Wolf et al., 2020). Depletion of Tregs leads to impaired clearance of very low-density lipoprotein cholesterol (VLDL -C) and celiac remnants, and increased atherosclerosis (Klingenberg et al., 2013). This depletion of Tregs leads to the transformation of the Treg cell phenotype and the subsequent loss of their immunosuppressive function (Gaddis et al., 2018). Similarly, this regulatory mechanism may attenuate IL-17-mediated atherosclerotic disease (Roy et al., 2022) Undoubtedly, atherosclerosis is a multifactorial disease characterized by the accumulation of numerous insults and immune-related factors, which can disrupt the normal homeostatic properties of the endothelial monolayer (Libby et al., 2019; Libby, 2021). Local accumulation of cholesterol has an important impact on the development of atherosclerosis, especially low-density lipoprotein cholesterol (LDL-C), which triggers an immune response in CD4^+^ T-helper cells (Th).

Cholesterol accumulation in cellular lipid rafts promotes the activation and proliferation of T cells as well as the differentiation of Th1 and Th17 cells (Chistiakov et al., 2019). HDL cholesterol was positively related to the relative frequency of conventional Tregs (CD25++CD127−cells in CD4^+^CD45RA−T cells). LDL-C levels were negatively correlated with the number of conventional Treg (Schmitz et al., 2023).

DEL-1 increases the expression of runt-related transcription factor-1 (RUNX1) and FOXP3 in conventional T cells, facilitating their conversion to induced Treg (Li et al., 2020). In the absence of exogenous TGF-β1, the stability of FOXP3 expression conferred upon Treg restimulation has been associated with reduced recruitment of inflammatory cells and decreased pro-inflammatory cytokine production, which is strongly linked to cardiovascular health (Li et al., 2020; Failer et al., 2022). Intriguingly, Treg cells can also transform proatherogenic subsets, including CD8^+^ T cells and γδ T cells. However, the mutual crosstalk between T-cell subsets and different types of immune cells has not been clarified (Saigusa et al., 2020). In addition, previous studies did not specifically regulate DEL-1 or further investigate the downstream pathways or mechanisms.

### 2.4 Macrophage-derived DEL-1 regulates efferocytosis

Previous studies have indicated that DEL-1 inhibits the production of macrophage migration inhibitor (MIF) in macrophages and also suppresses nuclear factor kappa-B (NF-κB)-mediated downstream inflammatory activation (Lee et al., 2014).

Efferocytosis, the process of removing dying cells, signifies the end of programmed cell death. Apoptotic cells are effectively phagocytosed and cleared through increased secretion of anti-inflammatory mediators and the activation of reparative transcriptional programs (Doran et al., 2020). Through the enzymatic action of scramblase, phosphatidylserine is exposed on the outer membrane of these dying cells, as a critical signal for phagocytes to recognize and engulf apoptotic cells (Leventis and Grinstein, 2010; Segawa and Nagata, 2015). Efferocytosis is vital for maintaining vascular homeostasis and health, as it inhibits inflammation and attenuates the progression of lesions such as atherosclerosis and aortic dissection (Zhang et al., 2019; Doddapattar et al., 2022; De Meyer et al., 2024). During efferocytosis, apoptotic cell-derived nucleotides promote the proliferation of noninflammatory macrophages, thereby facilitating inflammation regression and establishing a regenerative feedback loop (Gerlach et al., 2021). Disruption of this positive feedback loop results in a decline in phagocytosis and secondary efferocytosis, causing secondary necrosis in apoptotic cells and the release of intracellular DAMPs (Nagata, 2018; Morioka et al., 2019). Deficient efferocytosis, as a sign of inadequate inflammation resolution, leads to the accumulation of secondarily necrotic macrophages (Kasikara et al., 2021). Enhancement of efferocytosis has potential therapeutic implications for the maintenance of vascular integrity (Lee et al., 2018).

Macrophage-derived DEL-1 aids in resolving inflammation by increasing efferocytosis. Specifically, the αvβ3 integrin on macrophages mediates the efferocytosis of apoptotic cells (Kourtzelis et al., 2019). Upon exposure to external pathogenic factors, macrophages may attenuate the DEL-1-induced efferocytosis and related downstream elements within macrophages themselves (Li et al., 2023a). DEL-1 has been implicated in several cellular signaling pathways related to efferocytosis. Sirtuins (SIRTs) are a family of NAD+-dependent deacetylases, including histone deacetylases that regulate glucose homeostasis, inflammation, genome stability, and DNA repair (Chang and Guarente, 2014; Liu et al., 2021). SIRT6 regulates macrophage infiltration and inflammation resolution by targeting the miR-216/217 cluster in the DEL-1/CD36 axis and contributes to the resolution of inflammation in diabetic periodontitis (Zhao et al., 2021). DEL-1 also exhibits promise in the upstream regulation of efferocytosis, which is mediated by SIRT6 (Grootaert et al., 2021; Li et al., 2022c). It promotes efferocytosis to enhance the resolution of inflammation and has been shown to alleviate hypertension-induced fibrosis, medial thickening, and elastin loss in the aortic epicardium through its immunomodulatory effects (Dasgupta et al., 2012b; Failer et al., 2022).

To sum up, the expression of DEL-1 promotes macrophage efferocytosis and the elimination of apoptotic cells and prevents the activation of the immune response (Yatim et al., 2017). Efferocytosis promotes macrophage production of vascular endothelial growth factor C (VEGFC) and effectively inhibits pro-inflammatory macrophage activation (Glinton et al., 2022). Endothelial cells can produce vasoactive substances to alter or maintain hemodynamics (Kruger-Genge et al., 2019).

The protein PCSK9 is implicated in accelerating vascular aging by hindering efferocytosis in endothelial cells. This indicates that efferocytosis may play a crucial role in slowing down vascular aging (Liu et al., 2023). Although the role of DEL-1 in promoting efferocytosis in non-macrophagic cells has not been established, research is required to confirm whether DEL-1 is involved in efferocytosis in endothelial cells, as endothelial cells are one of the significant producers of DEL-1 (Hidai et al., 1998). This could help to identify the potential protective mechanisms of DEL-1.

## 3 The enigma of DEL-1 in atherosclerosis

DEL-1 may promote atherosclerosis, contrary to earlier descriptions of its anti-inflammatory and efferocytosis effects (Hajishengallis and Chavakis, 2019). Surprisingly, DEL-1 expression was predominantly localized to foam cells within the intimal layers of coronary arteries in patients with coronary heart disease (CHD) and sudden cardiac death (SCD) and was significantly elevated compared to that in healthy people. In ApoE^-/-^ mice, a positive correlation was observed between macrophage-derived DEL-1 levels and various atherosclerosis-related factors, such as plaque area, luminal stenosis, plaque foam cell counts, and downstream inflammatory factors. Moreover, the inhibition of the upstream pathway resulted in the suppression of DEL-1 expression, thereby exacerbating atherosclerotic lesions (Lu et al., 2023). This finding underscores the potential significance of DEL-1 in the regulation of atherosclerosis and warrants further investigation of its role in atherosclerosis. Previous studies have indicated that DEL-1 inhibits the production of macrophage migration inhibitor (MIF) in macrophages and also suppresses nuclear factor kappa-B (NF-κB)-mediated downstream inflammatory activation. DEL-1 is implicated in cardiovascular pathology through contrasting roles; while it appears to confer protection against inflammatory processes and vascular damage, recent evidence shows it may be associated with the promotion of atherosclerosis (Lee et al., 2014).

Chronic local inflammatory dysregulation may disrupt the regulation of endothelial immunity, such as enhanced vascular permeability, which regulates the recruitment of white blood cells and the activation of immune cells, may be impaired (Lee et al., 2014; Wang et al., 2022a). Instead, macrophages exert their pro-inflammatory effects after foam cell formation during atherosclerosis (Rader and Puré, 2005; Li et al., 2022a). The study mentioned above did not investigate the effects of the specific regulation of DEL-1 on downstream signaling, and there may be non-redundant signals other than DEL-1 that play a cross-regulatory role, thus affecting the results. However, previous research has shown that DEL-1 can bind to the phospholipid structure of oxidized low-density lipoprotein (oxLDL) and can inhibit the uptake of oxLDL by human coronary artery endothelial cells (HCAECs) or macrophages in a dose-dependent manner (Kakino et al., 2016). DEL-1 also inhibits the oxLDL-induced expression of monocyte chemoattractant protein-1 (MCP-1) and ICAM-1, as well as the secretion of endothelin-1 by HCAECs, ultimately mitigating atherosclerosis (Bennett et al., 2016; Kakino et al., 2016). The impact of the mice’s age on the experimental results has not yet been determined. There are methodological differences between the use of different drugs in animal models of atherosclerosis that may contribute to discrepancies in research findings related to the role of DEL-1 in atherosclerosis (Kakino et al., 2016; Emini Veseli et al., 2017). Given the variance in findings, it is critical to verify the physiological role of DEL-1 in atherosclerosis using a variety of animal models to ensure the consistency and reproducibility of results.

Additionally, overexpression of DEL-1 under atherosclerotic conditions may only be a feed-forward mechanism closely related to the complex inflammatory state of the disease and is not sufficient to suggest that DEL-1 promotes atherosclerosis (Di Pietro et al., 2023). DEL-1 effectively regulates the recruitment and chemotaxis of leukocytes, thereby preventing excessive inflammation (Hajishengallis and Chavakis, 2019). This is an important mechanism of its potential therapeutic effect.

## 4 DEL-1 facilitates angiogenesis

Angiogenesis, the process of forming new blood vessels, is driven by signals originating from surrounding tissues (Ramasamy et al., 2015; Jeong et al., 2021). Endothelial cells not only participate in this process by releasing proangiogenic growth factors but also exhibit dramatic plasticity, regulating vascular shape and organization through autocrine and paracrine signaling mechanisms (Bentley et al., 2014; McCoy et al., 2021). They can release inflammatory mediators, cell adhesion molecules, and immunomodulatory molecules to regulate normal vascular physiology (Shao et al., 2020). When endothelial cells are exposed to pathogens or inflammatory stressors, they may undergo a cascade of maladaptive changes including disruption of glycolytic and lipoprotein pathways, thereby contributing to vascular diseases (Xu et al., 2021).

DEL-1 is notable for its role in promoting angiogenesis, which is the formation of new blood vessels. The protein is actively expressed by embryonic endothelial cells where it contributes to the intricate shaping and remodeling of the developing vasculature, illustrating its importance during early growth stages (Hidai et al., 1998). DEL-1-induced angiogenesis is dependent on the integrins αvβ3 and αvβ5, in addition to some forms of embryonic and tumor-induced angiogenesis (Zhong et al., 2003). Studies have demonstrated that DEL-1 can interact with vascular endothelial growth factor (VEGF) to regulate its role in angiogenesis (Zou et al., 2009; Niu et al., 2023). This interaction may have the potential to prevent aortic dissection by increasing VEGF pathway inhibitors (Oshima et al., 2017).

Evidence suggests that DEL-1 (whether administered or overexpressed) contributes to angiogenesis, and a lack of endogenous DEL-1 has not been shown to impact normal angiogenesis in animal models (Ho et al., 2004). The aortic ring assay showed that neither endothelial angiogenesis nor developmental angiogenesis in the retina was affected by DEL-1 deficiency (Klotzsche-von Ameln et al., 2017). This suggests that constitutive DEL-1 expression does not promote physiological angiogenesis (Hsu et al., 2008). It has been suggested that DEL-1 prevents ischemia-driven neovascularization by inhibiting inflammatory activation downstream of αLβ2 (Klotzsche-von Ameln et al., 2017). The functional differences exhibited by DEL-1, may be influenced by environmental factors. In the disc angiogenesis system, DEL-1 was shown to upregulate the expression of fibroblast growth factor and significantly enhance fibrovascular growth (Ho et al., 2004). Clinical investigations into DEL-1’s therapeutic applications have shown promise, with the administration of DEL-1 plasmids leading to improved clinical manifestations in patients suffering from peripheral arterial disease, though the improvements did not achieve statistical significance, which warrants further study to conclusively determine efficacy (Grossman et al., 2007).

An increase in endothelial expression of DEL-1 can promote angiogenesis and endothelial cell migration (Beckham et al., 2014; Cobb et al., 2021). Cohort studies of patients with uterine cancer have shown that an increase in EDIL3 in dermal mesenchymal stem cells (DMSCs) stimulates the expression of αvβ3 and α5β1 from endothelial cells and promotes endothelium-associated angiogenesis (Niu et al., 2022a; Niu et al., 2022b). These changes were not limited to physiological conditions. DEL-1 expression levels in psoriatic DMSCs are 2.54-fold higher than those in healthy controls (Niu et al., 2016). This activation results in excessive angiogenesis and vasodilation, ultimately leading to an increase in both epidermal thickness and microvascular density (Niu et al., 2020; Niu et al., 2022b). DEL-1 can promote vascular repair and neovascularization, leading to adverse remodeling. DEL-1 also interacts with αvβ6 integrin, which is a known activator of transforming growth factor-beta (TGF-β), and effectively inhibits integrin-mediated TGF-β activation. Interestingly, the DEL-1-mediated inhibition of TGF-β signaling only occurs in response to increased levels of αv integrins (Kim et al., 2020). Specifically, DEL-1 mediates the activation of TGF-β and ERK signaling, promoting epithelial-mesenchymal transition (EMT) and angiogenesis, a critical step that can contribute to the distant metastasis of tumor cells in hepatocellular carcinoma (Xia et al., 2015; Jeong et al., 2017).

The angiogenic effects of DEL-1 promote distant metastasis of tumor cells. However, DEL-1 inhibits inflammatory cell migration, thereby reducing associated tumor complications (Hyun et al., 2020). Further molecular and clinical research is crucial to determine the potential role of DEL-1 in the pathophysiology and treatment of peripheral vascular disease. It is also important to investigate whether uncontrolled neovascularisation and adverse vascular remodeling might occur.

## 5 DEL-1 in the clearance of platelets

Serving as versatile effectors in both thrombosis and hemostasis, platelets orchestrate immune responses by releasing pro-inflammatory cytokines, including IL-1β, thereby enhancing endothelial permeability and guiding other immune cells to areas of injury or infection (Pober and Sessa, 2007; Koupenova et al., 2018; Margraf and Zarbock, 2019).

Platelet microparticles (PMPs) are tiny fragments of cells that are released from platelets, which can be triggered by different stimuli, such as the activation of platelets by thrombin, collagen, or other agonists. Platelet overactivation and PMPs play a key role in the pathogenesis of cardiovascular and autoimmune diseases (Lannan et al., 2014). The size of PMPs depends on the nature of cell activation stimuli (Ponomareva et al., 2017).

The adhesion of platelets to the activated endothelium is facilitated by increased expression of adhesion molecules. This process, along with the subsequent binding of platelets to leukocytes, is crucial for forming platelet-leukocyte aggregates. These aggregates are instrumental in atherogenesis, with ongoing platelet-leukocyte interactions being a central component of this pathology (May et al., 2007; Twarock et al., 2016; Pircher et al., 2019). In a high-glucose environment, PMPs markedly diminish endothelial nitric oxide (NO) bioavailability while concurrently amplifying reactive oxygen species (ROS) synthesis. This dual action exacerbates endothelial dysfunction, culminating in heightened permeability and cellular damage (Wang et al., 2019a).

DEL-1 binds to platelet phosphatidylserine and mediates endothelial clearance of platelet particles. Due to redundant mechanisms, depletion of DEL-1 does not impact coagulation under normal physiological conditions but leads to enhanced coagulation in disease states characterized by pathological increases in particulate production (Dasgupta et al., 2012a). DEL-1 can selectively disrupt the interaction between Mac-1 integrins on leukocytes and platelets, thereby inhibiting the thrombotic inflammatory injury that can arise, for instance, during myocardial infarction when platelet-leukocyte aggregates contribute to thrombosis (Kourtzelis et al., 2016). Romanidou et al. reported that in pregnant patients with thrombotic microangiopathy, circulating DEL-1 levels are significantly diminished and may represent a significant biomarker (Romanidou et al., 2023). Although the specific mechanism by which the reduction in circulating DEL-1 contributes to this type of widespread systemic thrombosis has not been identified at this time, the clearance of PMPs by DEL-1 and inhibiting the associated inflammation and thrombosis may hold therapeutic potential in preventing thrombosis or vascular injury associated with the pathological state.

## 6 DEL-1 suppresses endoplasmic reticulum stress

The endoplasmic reticulum (ER) plays a key role in the synthesis, folding, and structural modification of proteins in cells (Schwarz and Blower, 2016). The endoplasmic reticulum stress (ER stress) response is a key regulator of inflammation and cell death. The capability of the ER to fold proteins varies greatly between cell types. When misfolded proteins accumulate in the endoplasmic reticulum, the ability of the ER to correctly fold and posttranslationally modify proteins can be compromised, leading to substantial cellular dysfunction which can contribute to both genetic mutations and vulnerability to environmental stressors (Wiseman et al., 2022). To maintain normal protein folding homeostasis, the endoplasmic reticulum activates the unfolded protein response (UPR) (Ron and Walter, 2007). However, excessive production of UPR signaling molecules can be detrimental. Persistent engagement of the UPR signaling cascade within the endoplasmic reticulum can also lead to an accumulation of misfolded proteins, resulting in ER stress (Oakes and Papa, 2015).

Prolonged ER stress can lead to an increased protein load and, if unmitigated, can ultimately result in cell death (Marciniak and Ron, 2006). Currently, two primary approaches target ER stress: one involves directly influencing the accumulation of misfolded proteins within the ER. While the other modulates the signaling of the unfolded protein response (UPR) via ER stress sensors or enzymes that regulate their downstream effects (Marciniak et al., 2022). The UPR is regulated by three endoplasmic reticulum transmembrane sensors: protein kinase R-like endoplasmic reticulum kinase (PERK), inositol-requiring enzyme 1α (IRE1α), and activating transcription factor 6 (ATF6). These factors are crucial for mediating the UPR and coordinating the cellular response to ER stress (Yap et al., 2021). Overexpression of DEL-1 has been shown to attenuate LPS-induced oxidative stress (Li et al., 2022b). In addition, DEL-1 attenuated palmitate-induced and HFD-induced skeletal muscle ER stress and insulin resistance via SIRT1/SERCA2-mediated signaling (Sun et al., 2020).

**FIGURE 2:**
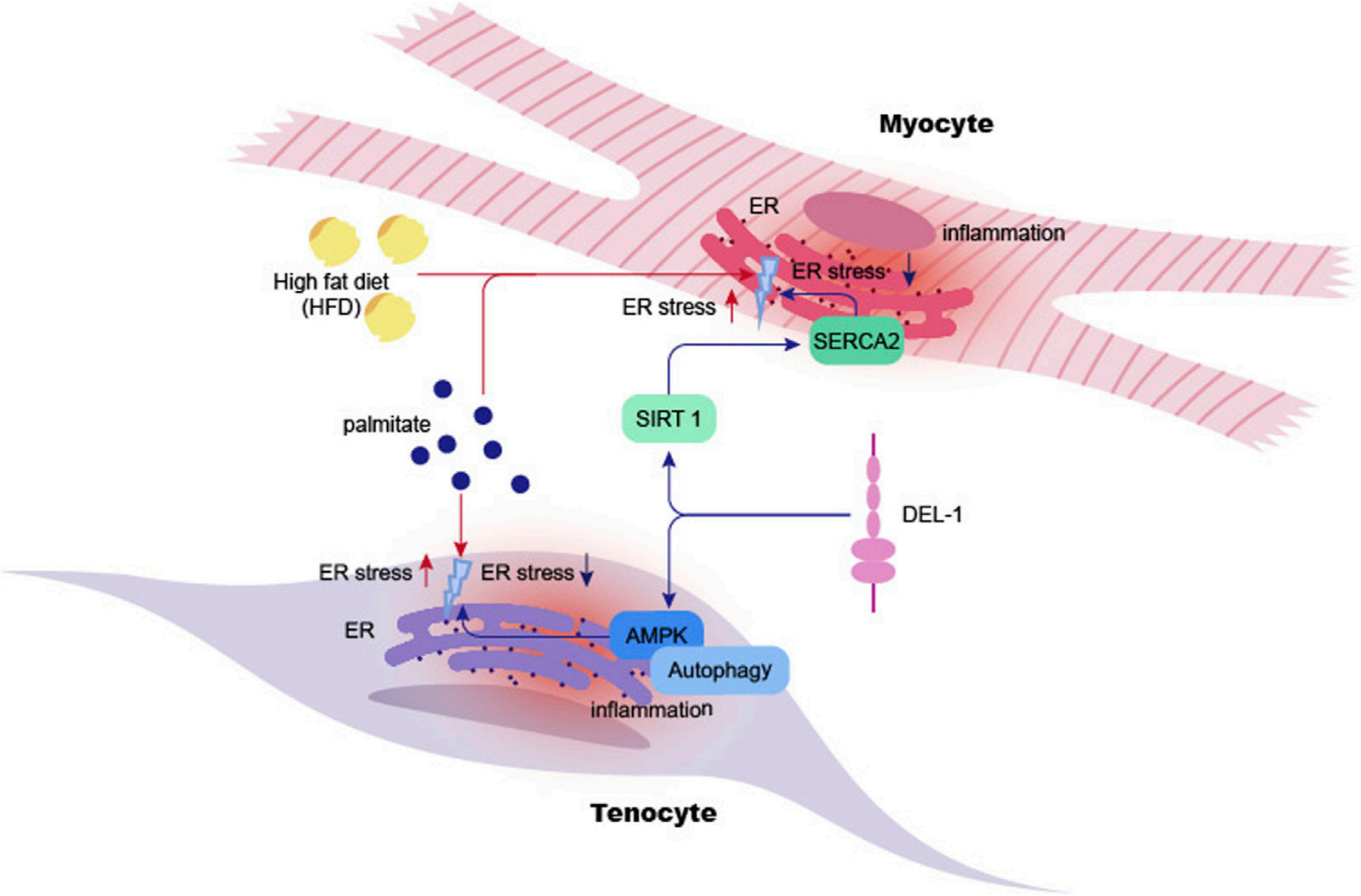
DEL-1 inhibits ER stress in myocyte and tenocyte. High-fat diet or palmitate significantly increases ER stress in myocyte cells in mice, leading to inflammation in myocytes and subsequent cellular damage. DEL-1 treatment enhances SIRT1 expression in myocytes in a dose-dependent manner, mitigating ER stress under palmitate treatment through the SIRT1/SERCA2 pathway. Additionally, DEL-1 enhances AMPK phosphorylation in tenocytes, initiating autophagy and reducing palmitate-induced inflammation. The figure indicates that DEL-1 helps alleviate muscle and tendon inflammation exacerbated by obesity. ER: Endoplasmic Reticulum, SIRT1: Sirtuin 1, SERCA2: Sarcoplasmic/Endoplasmic Reticulum Calcium ATPase 2, AMPK: AMP-activated protein kinase.

The AMP-activated protein kinase (AMPK) and peroxisome proliferator-activated receptor delta (PPARδ) are key regulatory proteins within the cell (Kemmerer et al., 2015). Downregulation of this signaling could promote ER stress-induced vascular endothelial dysfunction in adult rat offspring exposed to maternal diabetes (Luo et al., 2022). DEL-1 counteracts ER stress by activating the AMPK pathway, which, in turn, induces autophagy—a crucial defense mechanism that helps preserve cellular function. Additionally, DEL-1 protects tendon cells from stress-induced apoptosis (Park et al., 2022) (see Figure 2).

Within the vasculature, vascular smooth muscle is situated in the middle layer of the vessel and controls vasoconstriction and diastole. However, deviations from normal physiological activity in vascular smooth muscle can induce phenotypic transformation or diminish VSMC populations. Such changes can concurrently injure the endothelium, undermining vascular integrity (Baumann et al., 1981; Kuang et al., 2016; Grewal et al., 2021). Emerging evidence highlights that ER stress in VSMCs contributes to the progression of various vascular diseases through the release of extracellular vesicles and the disruption of autophagy; these diseases include vascular calcification and aortic dissection (Clement et al., 2019; Li et al., 2021b; Furmanik et al., 2021). In response to pathogenic stimuli, VSMCs may undergo phenotypic transformation into macrophage-like cells, a process often driven by the accumulation of lipids within atherosclerotic plaques (Bennett et al., 2016).

Mild or transient ER stress caused by the accumulation of misfolded or unfolded proteins, binding immunoglobulin protein (BiP) dissociates from IRE1α and ATF6 but remains bound to PERK. Then, IRE1α dimerizes and is phosphorylated, leading to the splicing of X-box binding protein 1 (XBP-1) (Cubillos-Ruiz et al., 2017). Notably, this factor inhibits pathological processes, including oxidative stress in endothelial cells and autophagy in VSMCs (Zhao et al., 2013; Martin et al., 2014). Concurrently, BiP dissociation triggers the translocation and cleavage of ATF6. If ER stress persists, BiP will also dissociate from PERK. This action induces the phosphorylation of the downstream protein eukaryotic translation initiation factor 2α (eIF2α), which is a crucial step in the ER stress response. This leads to the activation of the transcription factor C/EBP homologous protein (CHOP), which initiates the apoptotic pathway (Wu and Kaufman, 2006; Zhang et al., 2006; Cnop et al., 2017). In the case of atherosclerosis, prolonged and intensified ER stress contributes to sustained and enhanced UPR signaling, leading to cell death and exacerbating plaque formation in the arteries (Zhou and Tabas, 2013). Disturbed ER homeostasis triggers the progression of cardiovascular disease, which ultimately further exacerbates ER stress, creating a vicious circle (Ren et al., 2021).

Increased salivary gland CHOP expression in Sjögren’s syndrome (SS) is implicated in the promotion of apoptosis and in enhancing leukocyte infiltration. This upregulation correlates with decreased DEL-1 levels, providing mechanistic insight into the relationship between CHOP and DEL-1 (Baban et al., 2013). Inhibition of unspliced XBP1 (XBP1u) increases vascular calcification and stimulates ER stress to promote vascular remodeling. XBP1u interacts with β-catenin and promotes its degradation via the ubiquitin-proteasome pathway. This interaction inhibits the transcriptional activity of the beta-catenin/TCF complex, which is responsible for regulating osteogenic markers such as runt-related transcription factor 2 (Runx2) and Msh homeobox 2 (Msx2), which play roles in vascular calcification (Yang et al., 2022). When the autophagy protein 5 (Atg5) gene is absent in VSMCs, autophagy is impaired, increasing the susceptibility of VSMCs to cell death. This impairment in autophagy enhances ER stress activation, subsequently promoting inflammation in VSMCs through an IRE-1α-dependent mechanism (Clement et al., 2019). Cholesterol contributes to ER stress, which can trigger the conversion of VSMCs into cells exhibiting features characteristic of macrophages and fibroblasts, a process modulated by the UPR (Chattopadhyay et al., 2021).

Available evidence suggests that DEL-1 contributes to the inhibition of ER stress-induced apoptosis (Park et al., 2022). Recently, DEL-1 levels have been observed to be elevated in response to exercise, by inhibiting ER stress. This inhibition can contribute to reduced inflammation and improved insulin sensitivity, as indicated by evidence (Kwon et al., 2020). However, to date, no specific research has explored the ability of DEL-1 to alleviate ER stress in VSMCs. It is important to note that the ER in skeletal muscle is referred to as the sarcoplasmic reticulum (SR). Skeletal muscle fibers contain the sarcoplasmic reticulum, a highly differentiated structure composed of tubular networks aligned with sarcomeres, reflecting distinct morphological and structural characteristics compared to vascular smooth muscle (Cusimano et al., 2009; Sorrentino, 2011). In contrast, the ER in vascular smooth muscle, while lacking the specialized alignment of the SR, presents a reticulated network that pervades the entire cell. Investigating whether these structural differences influence the outcomes of DEL-1 intervention in VSMCs could provide valuable insights for therapeutic strategies. In addition to mitigating ER stress, DEL-1 may also protect against damage to VSMCs induced by other signaling pathways. For instance, signaling by he receptor activator ofnuclear factor kappa-beta ligand (RANKL) and the cytoplasmic calcineurin-dependent nuclear factor of activated T-cells (NFATc1) has been shown to facilitate the upregulation of bone morphogenetic proteins (BMPs) (Panizo et al., 2009; Singh et al., 2012; Shiny et al., 2016). Furthermore, DEL-1 has been demonstrated to inhibit the RANK-NFATc1 signaling axis, thus impeding osteoblast activation (Shin et al., 2015). This interaction likely represents a crucial mechanism by which DEL-1 sustains vascular integrity and health. The paracrine signaling between endothelial cells (ECs) and vascular smooth muscle cells (VSMCs) is likely to play an instrumental role in mediating the vasoprotective effects of DEL-1.

## 7 DEL-1 as a potential therapeutic target and outlook in vessels

In various vascular diseases, inflammation is a crucial factor in promoting a spectrum of pathological changes (An et al., 2019). DEL-1 regulates the production of inflammatory factors and the activation of signaling pathways, thereby attenuating the impact of immune cells and their associated inflammation on the vasculature (Li et al., 2021a). The current evidence on the therapeutic potential of DEL-1 for cardiovascular disease remains a subject of debate. This controversy highlights the necessity for more rigorous clinical studies and mechanistic research to clarify the therapeutic benefits of DEL-1 in cardiovascular pathology (Zhao et al., 2022).

**FIGURE 3:**
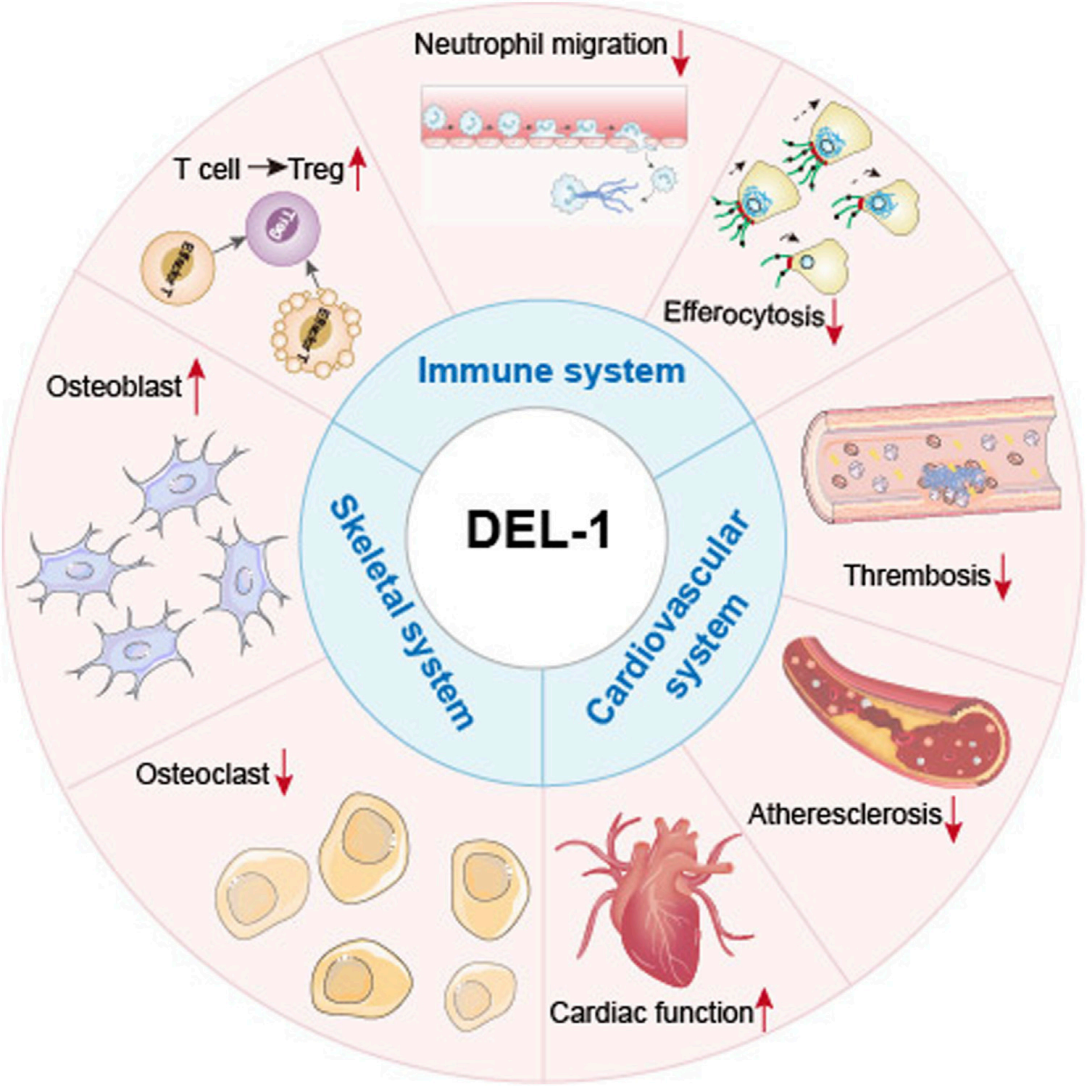
General roles of DEL-1 in various systems and cell types. Summary of the physiological roles of DEL-1 in various organs or cells within the cardiovascular, immune, and skeletal systems. In the cardiovascular system, DEL-1 improves cardiac function and inhibits thrombosis and the progression of atherosclerosis. In the immune system, DEL-1 exerts immunomodulatory functions through interactions with various integrins, including inhibiting immune cell activation processes such as neutrophil migration; enhancing the efferocytosis process of phagocytes to promote inflammation resolution; promoting the differentiation of other T cell types into Treg cells, and inhibiting the excessive activation and proliferation of effector T cells. In the skeletal system, DEL-1 promotes the maturation of osteoblasts, inhibits the maturation of osteoclasts, promotes bone regeneration, and reduces inflammatory responses.

Further exploration of DEL-1 as a potential clinical therapeutic target or biomarker for vascular diseases is warranted. The differences in the expression and function of DEL-1 in different organs suggest that assessing its viability as a therapeutic target could be an important direction for future research (Figure 3). Amidst the ongoing debate on the use of DEL-1, its capacity to suppress inflammation and cytotoxicity should be acknowledged as a therapeutic option for cardiovascular diseases, targeting multiple cell types including ECs and VSMCs (Wang et al., 2022b). Further research is required to develop treatment modalities or drug delivery methods. This would allow the investigation of crosstalk between DEL-1 and other regulators of angiogenesis, thereby advancing our understanding of the role of DEL-1 in cardiovascular disease treatment (Cabrera and Makino, 2022; Zhao et al., 2022).

Conditional Knockout of the EDIL3 gene using CRISPR/Cas9 gene-editing technology may help assess the effects of DEL-1 on downstream signaling molecules byreducing the interference from any compensatory mechanisms or indirect signaling pathways, assessing functional changes in the intima, media, and adventitia layers of the vasculature (Wang et al., 2022b; Li et al., 2023b). The immunomodulatory effects of DEL-1 vary among cell types and environments, highlighting its immune plasticity. Although a connection has been identified between DEL-1 and specific vasoactive factors in angiogenesis (Niu et al., 2023), the interplay between DEL-1 and other molecules remains poorly understood. As vascular tissue engineering moves toward clinical applications, replicating vascular physiological function is one of the key barriers (Ciucurel and Sefton, 2014; Margolis et al., 2023). There have been limited studies exploring the use of DEL-1 in tissue engineering constructs to regulate endothelial cells (Ciucurel and Sefton, 2014). Looking forward, endogenous factors like DEL-1 mayprove crucial for the fabrication of a multiscale vascular hierarchy under physiological conditions. While DEL-1 has shown promising safety results in a clinical study (Grossman et al., 2007), our incomplete understanding of its physiological functions underscores the need for refined animal experiments to comprehensively determine its effects on various systems before advancing to further clinical trials.
